# Validation of a Liquid Biopsy Protocol for Canine BRAFV595E Variant Detection in Dog Urine and Its Evaluation as a Diagnostic Test Complementary to Cytology

**DOI:** 10.3389/fvets.2022.909934

**Published:** 2022-05-31

**Authors:** Fabio Gentilini, Christopher J. Palgrave, Michal Neta, Raimondo Tornago, Tommaso Furlanello, Jennifer S. McKay, Federico Sacchini, Maria E. Turba

**Affiliations:** ^1^Department of Veterinary Medical Sciences, University of Bologna, Ozzano dell'Emilia, Italy; ^2^IDEXX Laboratories Ltd., Wetherby, West Yorkshire, United Kingdom; ^3^Veterinary Clinic and Laboratory San Marco, Veggiano, Italy; ^4^Genefast srl, Forlì, Italy

**Keywords:** somatic mutation, oncogenes, histopathology, precision medicine, BRAF, liquid biopsy

## Abstract

A significant proportion of canine urothelial carcinomas carry the driver valine to glutamic acid variation (V595E) in BRAF kinase. The detection of V595E may prove suitable to guide molecularly targeted therapies and support non-invasive diagnosis of the urogenital system by means of a liquid biopsy approach using urine. Three cohorts and a control group were included in this multi-step validation study which included setting up a digital PCR assay. This was followed by investigation of preanalytical factors and two alternative PCR techniques on a liquid biopsy protocol. Finally, a blind study using urine as diagnostic sample has been carried out to verify its suitability as diagnostic test to complement cytology. The digital PCR (dPCR) assay proved consistently specific, sensitive, and linear. Using the dPCR assay, the prevalence of V595E in 22 urothelial carcinomas was 90.9%. When compared with histopathology as gold standard in the blind-label cases, the diagnostic accuracy of using the canine BRAF (cBRAF) variation as a surrogate assay against the histologic diagnosis was 85.7% with 92.3% positive predictive value and 80.0% negative predictive value. In all the cases, in which both biopsy tissue and the associated urine were assayed, the findings matched completely. Finally, when combined with urine sediment cytology examination in blind-label cases with clinical suspicion of malignancy, the dPCR assay significantly improved the overall diagnostic accuracy. A liquid biopsy approach on urine using the digital PCR may be a valuable breakthrough in the diagnostic of urothelial carcinomas in dogs.

## Introduction

According to the somatic mutation theory, cancer is linked to acquired “driver” mutations which trigger and sustain carcinogenesis (1). Mutations in the mitogen-activated protein kinase (MAPK) pathway, also known as the RAS/RAF/ERK/MEK pathway, are found in many cancer types. The MAPK pathway is important in promoting cell growth, proliferation, and survival (2, 3). Activating mutations within the pathway or overexpression of the pathway lead to uncontrolled cell growth. The V600E mutation in BRAF, one of the MAPK genes, is an important driver mutation in a variety of cancers. Most notably, more than 60% of human thyroid adenocarcinomas (4) and more than 50% of human cutaneous melanomas (5) carry the BRAF V600E mutation (https://portal.gdc.cancer.gov/genes/ENSG00000157764). Direct targeting of the mutant gene products and their respective pathways, regardless of tumor type is an example of precision medicine (6, 7).

Urothelial carcinomas (UCs) are common tumors in both humans and dogs, representing 1-2% of all naturally occurring cancers in the latter (6, 8–11). Considerable evidence suggests that invasive UC (invUC) in humans shares many similarities with its canine counterpart, making it a good translational animal model (9, 12). Similarities include epidemiology, clinical signs, management and therapy, in addition to macroscopic, histopathological and molecular features (12, 13). A significant proportion, ranging from 44.6 to 87.9% (8, 14, 15), of canine UC, formerly called transitional cells carcinoma (TCC) carries the valine (V) to glutamic acid (E) variation as a result of a substitution in the second position of codon 595 (V595E) in canine BRAF (cBRAF) kinase, which is homologous to theV600E mutation in humans (8). In contrast, human invUC rarely has BRAF mutations; however, approximately one third of the tumors carry somatic mutations in other genes in the MAPK pathway (6, 9, 12, 13, 16).

The discovery of the high prevalence of the cBRAF V595E driver mutation in canine UC has two main implications; the first is the identification of a potential therapeutic target (17–19) and the second is the possibility of developing liquid biopsy protocols, i.e., non-invasive procedures for diagnosing canine UC (8). Traditional diagnostic sampling of the urogenital system can be invasive and may require biopsy; however, the identification of BRAF mutations in urine and cytological smears prepared from urine sediment or the plasma of affected patients would provide minimally invasive diagnostic techniques (14, 15, 20). Furthermore, the BRAF mutation seems to be an early event and its prompt detection in routine samples could be an early diagnostic tool for rapid intervention. Liquid biopsy is clearly required, but achieving an accurate result is challenging. Many factors, either preanalytical (such as the matrix used for genomic DNA (gDNA) purification and the method of purification) or analytical (such as the technique used), can affect the accuracy. To date, both quantitative PCR (qPCR) (15, 20) and droplet digital PCR (ddPCR) (14) have been utilized.

Digital PCR (dPCR) is a relatively recent evolution of PCR technology with some practical advantages over standard qPCR assays. Specifically, dPCR allows for the absolute quantitation of nucleic acid samples without the need for calibration curves based on the amplification of single template molecules. Digital PCR is inherently more sensitive, specific and precise than standard qPCR, and is particularly suitable for the detection of rare cancer targets (21). Many dPCR platforms exist, featuring different systems of partitioning: microfluidic-chambers, droplets obtained by oil-water emulsion and micro-well chip-based. Despite inherent variations in the platforms, their performance in terms of sensitivity, specificity, and precision appears to overlap (22). Although it is expected that dPCR performs better than the qPCR, no direct comparison is available between these techniques in detecting cBRAFV595E. It would be relevant to quantify the gap in term of diagnostic performances on equal terms.

This study was primarily aimed at setting up and evaluating a dPCR assay for detecting the cBRAFV595E variant. Secondly, it aimed to establish the best combined protocol for detecting cBRAF V595E pathogenic variants by investigating critical preanalytical factors, such as matrix and nucleic acid purification protocols, in addition to the different PCR techniques. Finally, using a diagnostic cohort study, the cBRAF liquid biopsy protocol findings were compared to the traditional cytological examination of urine sediment in order to establish its role, if any, in supporting the diagnosis of UC in dogs.

## Materials and Methods

### Experimental Design and Samples

**Figure 1:**
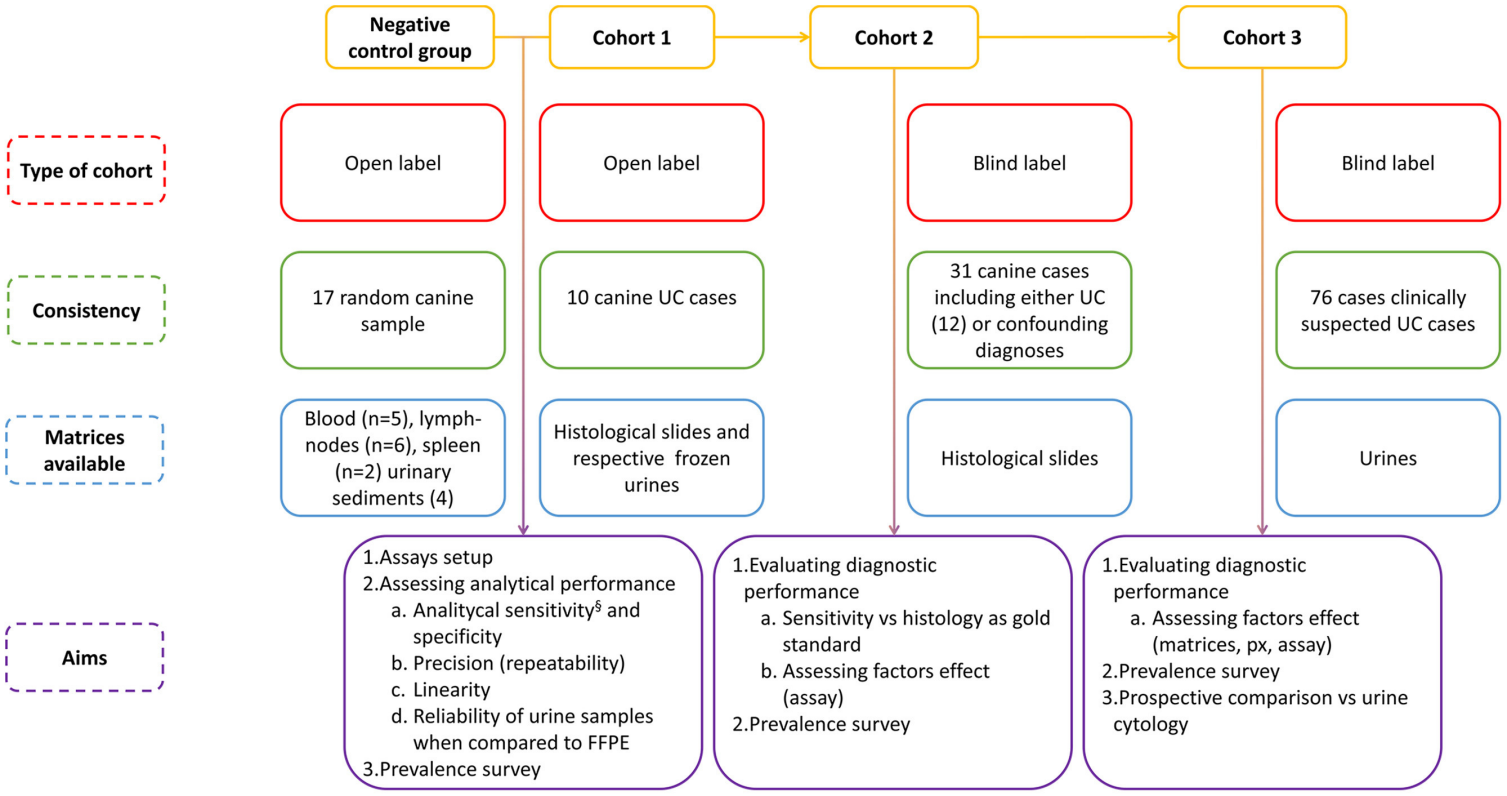
Schematic representation of the experimental layout^§^ which also includes samples from cohorts 2 and 3.

The experimental layout is summarized in Figure 1. The study included three cohorts of samples known to carry, or suspected of carrying, the cBRAF V595E variant, together with a heterogeneous group of samples (negative control group) previously used for molecular diagnostic testing other than cBRAF. The first cohort, used for developing and validating the method, consisted of 10 open-label cases previously diagnosed with UC of the bladder. Histological sections from biopsies collected endoscopically or by surgical excision, and the respective whole frozen urine samples (Supplementary Table 1), collected over a period of 10 years, were retrieved from the repository.

The second cohort was represented by a larger group of blind-label histological slides taken from biopsies of cases submitted with a clinical suspicion of UC over a period of 10 months. The vast majority were from the urinary tract; a small number were from other sites. This cohort consisted of 31 samples, and the molecular biologists (MET, FG) were unaware of the diagnosis. For statistical purposes, the diagnoses were categorized as malignant UCs (N = 12), cystitis (N = 3), polyps (N = 3), polypoid cystitis (N = 3), hyperplasia/dysplasia/benign lesions (N = 3) and other carcinomas (N = 7) (Supplementary Table 1). The dogs in cohorts 1 and 2 had formalin-fixed paraffin embedded (FFPE) slides archived. Histological slides were used to obtain genomic DNA (gDNA) for molecular analysis. No histological blocks were used in any case. The tissue samples had been preserved in 10% neutral-buffered formalin and allowed to fix for at least 24 h. The samples were processed, embedded in paraffin wax, sectioned and stained with hematoxylin and eosin for routine histopathological examination by a board-certified anatomic pathologist.

The third cohort was the largest and consisted of urine-derived samples prospectively included in the study due to suspected neoplasia in the urinary tract, based on clinical signs and/or ultrasonographic findings and/or cytological examination of the urinary sediment carried out by board-certified clinical pathologists. This group consisted of 76 cases, all of which included the urine sediment and, in 47 cases, also the respective urine supernatant. In this group, the diagnosis was presumptive. The cytological reports (presumptive diagnoses) were categorized for statistical purposes as unrewarding findings, inflammation, haematuria, pyuria, epithelial atypia (including dysplasia), likely malignancy, overt malignancy (and possible origin); the latter three were additionally categorized as likely neoplastic and the former likely non-neoplastic for statistical purposes (Supplementary Table 1). The urine samples used in the study were collected by veterinarians in different ways (free catch, cystocentesis and by means of a catheter). The samples were sent to the diagnostic laboratory where they were centrifuged at 1,600 rpm for 5 mins. The supernatant was removed and transferred to a separate tube. The pellet was mixed using a vortex mixer and was used to make cytological smears. The smears were either used for molecular evaluation or stained with Modified Wright's Giemsa and cover slipped for cytological examination by a board-certified clinical pathologist.

The group of heterogeneous gDNA samples, (negative control group) composed of gDNA which was highly unlikely to have the cBRAF variant, was made up of blood samples (*N* = 5), lymph node aspirates or other cytological smears (*N* = 5), and from histological slides of the spleen (*N* = 2) or lymph nodes (*N* = 1) or urine sediment (*N* = 4). This group of 17 samples was used for evaluating the specificity of the assays.

### DNA Purification

The isolation of gDNA is a critical point in the workflow for the molecular detection of BRAF V595E. In the present study, all the samples, regardless of the different matrices examined, were purified using an automated extractor (Maxwell RSC 48, Promega) which relied on a magnetic particle mover and paramagnetic beads; the instrument can run different cartridge-based methods suitable for each specific matrix. The Maxwell RSC DNA formalin-fixed, paraffin-embedded (FFPE) Kit was used to purify the gDNA from 41 FFPE tissue samples taken from the respective histological slides; The Maxwell RSC Blood DNA Kit was used to purify the gDNA from 76 urinary sediments and also from 23 urine supernatants and the Maxwell RSC circulating cell-free DNA (ccfDNA) plasma kit was used to extract the gDNA from 46 urine supernatant (Supplementary Table 1).

The methods of purification from the negative control group of samples included either the FFPE or the blood DNA kit. All the methods were carried out according to the protocols run on the automated instrument.

### QPCR and dPCR

The qPCR assays were carried out on CFX Connect or CFX96 Touch thermal cyclers (Biorad) while the dPCR assays were carried out on the QuantStudio 3D Digital PCR system (Thermo Fisher), using the protocols reported in the Supplementary Material.

### Analytical Performances of dPCR BRAF Assays

The analytical performances of the dPCR assays were established in terms of analytical sensitivity, precision and linearity, and were expressed as Limit of Detection (LOD) and Coefficient of Variation (CV %), and as a linear coefficient of correlation R^2^, respectively. To estimate the LOD, the results of 17 negative samples and 5 replicates of 7 positive samples with less than 1% of mutated allele were analyzed, and the LOD was estimated by fitting a Probit regression model with a prediction level of 95%.

Linearity was evaluated using serial dilutions of positive gDNA, either in terms of absolute concentration (by diluting the positive sample in molecular biology grade water) separately for each fluorophore or in terms of the percentage of a mutated allele with a majority of wild-type targets (by diluting the positive samples in a wild-type gDNA sample). The results were analyzed using a linear regression model.

To evaluate precision as inter-assay repeatability, positive samples were stratified based on the fraction of the BRAF variant found in the samples in low (<1%) medium (>1% and < than 10%) and high (>10%) percentages. Three samples in each category were then re-assayed on separate days by repeating both PCR and chip reading. The CVs across the technical replicates were calculated as Standard Deviation/Mean × 100, both for all categories together and for each category separately.

To establish the analytical specificity, the dPCR assay was used to analyse the gDNA purified from a range of samples previously analyzed for reasons other than BRAF testing and which were considered unlikely to be positive for the *BRAF* mutation. Matrices from which the gDNA was purified included the spleen, lymph nodes, urine sediment and blood samples (*n* = 17).

Finally, to evaluate the reliability of using urine instead of tissue from histological slides as a matrix for the purification of gDNA, the dPCR test results from gDNA obtained from the FFPE and from the respective urine samples of the open-label cohort 1 were compared (Figure 1).

### Diagnostic Accuracy

In order to optimize the workflow and to establish the best practices for the urine liquid biopsy protocol, various factors were evaluated. In particular, the following pre-analytical and analytical factors were considered: urine sediment or urine supernatant as a matrix for gDNA purification and, in the latter group, the standard Maxwell RSC Blood DNA or the Maxwell RSC ccfDNA plasma kit were used for purifying the circulating cell-free DNA. From the analytical perspective, the same genotyping assays were compared when used in qPCR and in dPCR. To evaluate the diagnostic accuracy of using the optimized cBRAF V595E protocol (UC liquid biopsy protocol) as a surrogate UC diagnostic tool, the V595E findings in blind-label cohort 2 were then compared with the histologic diagnosis which was considered to be the gold standard. Finally, the UC liquid biopsy protocol was prospectively applied to analyse the urines of a cohort of dogs (cohort 3) with clinical suspicion of UC, and the findings were compared with the cytology (Figure 1).

### Statistical Analysis

Diagnostic performance analysis and Bangdiwala agreement plots (23) were carried out using Microsoft Excel, including the Analyse-it package Software. The pathological and molecular findings of the blind cohorts were compared using descriptive statistics and a 2 × 2 contingency table with chi-square. A *P*-value < 0.05 was considered significant.

## Results

As a first step, a novel dPCR assay was set up and validated; the dPCR assay and the already described qPCR (15) assays were then used to assess a range of tissue, urine sediment and supernatant samples, the gDNA of which were purified using different methods.

### Analytical Performances

**Figure 2:**
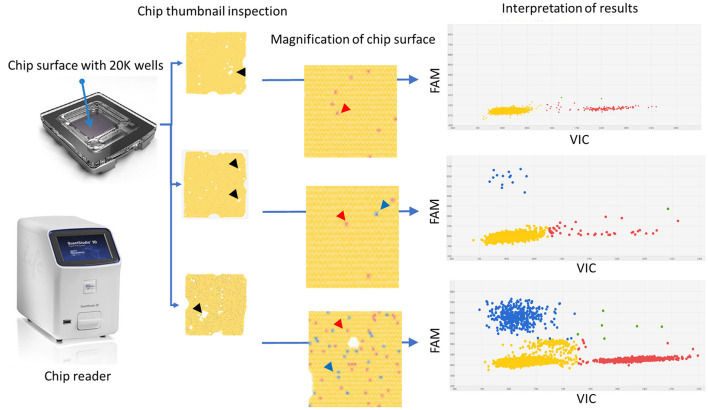
Graphical description of the digital PCR assay workflow including selected examples of results. On the left, the chip and chip reader instrument. The thumbnail appearance of the chip surfaces and their magnifications are reported. They are used to assess whether there is a homogeneous coverage of the chip surface and to rule out the presence of dust or debris or “bubble” or other event detrimental for the quality of the readings. White spots represent areas in which the reaction mix did not fill the wells (black harrow head). At high magnification wells could be identified. Each dot represents a well containing either “no fluorescence” as yellow dots (prevalent background) or “VIC fluorescence” as red dots (red arrow heads) or “FAM fluorescence” as blue dots (blue arrow heads). On the right, the plot two-axis graphical representation of the results. The X-axis reports the VIC fluorescence (wild-type target) while the y-axis reports the FAM fluorescence (mutated target); each dot represents a well containing fluorescence; the green dots and the yellow dots are either VIC and FAM positive wells or negative wells, respectively. Red dots and blue dots represent wells containing only VIC (wild-type target) or FAM (mutated target) fluorescence, respectively. On the right, from top to bottom: negative sample (only red dots and yellow dots), positive diluted sample (few positive wells with high percentage of mutated “target FAM”), and positive concentrated sample (many positive wells with high percentage of mutated “target FAM”). It is worth noting that only at high target concentrations do the clusters tend to overlap.

Interpretation of the results was intuitive and unequivocal since the two clusters of dots, specifically the “No amplification” cluster and the “FAM positive” cluster, were well distanced and not overlapping (Figure 2). Discrete VIC (mean ± SD: 1,107 ± 293; range: 349-1715) and FAM (mean ± SD: 2,950 ± 855; range: 1,073-4,667) threshold ranges were defined using the likely wild-type samples (open label, negative control group). Furthermore, in this group of samples, the dPCR assay did not amplify any off-target products (analytical specificity).

**Figure 3:**
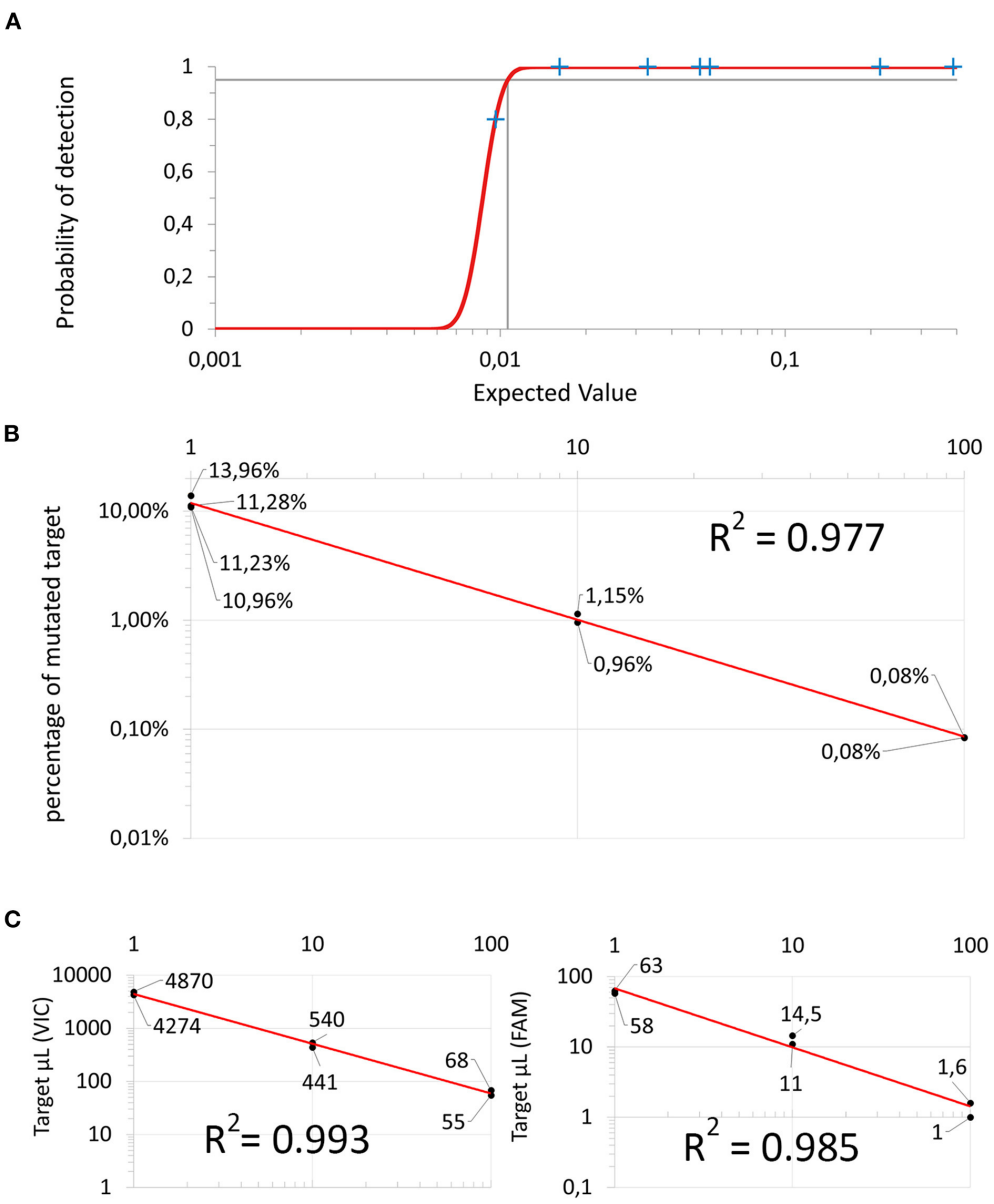
Dilution experiments: **(A)** Probit regression curve used to infer the Limit of Detection. **(B)** 1:10 serial dilution using a sample containing only wild-type “target VIC” to dilute mutated “target FAM”. **(C)** 1:10 serial dilution using molecular biology grade water. Both the wild-type “target VIC” on the left and the mutated “target FAM” (on the right) are linearly diluted. The X-axis represents the dilution factor and the y-axis represents the absolute amount of copies/μL of target.

In linearity experiments, both the percentage and the absolute target concentration showed a linear reduction in their value proportional to the dilution factor (Figures 3B,C). These results confirmed adequate efficiency of the amplification of the dPCR assay as a whole and for each specific probe component, the R^2^ coefficients being 0.977, 0.993, and 0.985, respectively.

The dPCR assay was able to reproducibly detect approximately one of the targets of 1 × 10^4^ wild-type targets (10^−4^). The LOD was estimated to be 0.011% (analytical sensitivity) (Figure 3A).

Inter-assay repeatability showed CVs of 14.0, 11.0, and 25.1% in the low, medium and high mutation fraction groups, respectively. Overall, the CV was 16.7% (repeatability, precision).

### Prevalence of the cBRAF Mutation

When considering both the blind and the open-label cohorts together, the cBRAF V595E variation was found in 20 of the 22 cases (90.9%). All ten of the open diagnosis cases were positive for cBRAF using either FFPE purified DNA from histological slides or CCF purified DNA from matched frozen whole urine. In the second cohort, the cBRAF variant was also found in 2 of the 3 prostatic carcinoma and 1 of the 2 prostatic hyperplasia samples. Of the other carcinomas investigated, the cBRAF variant was found in a metastatic carcinoma, a nasal transitional cell carcinoma, a nasal adenocarcinoma and a penile squamous cell carcinoma. The sole papillary urothelial neoplasm with low malignant potential did not carry the cBRAF variant.

### Diagnostic Performance

In the second cohort using a blind approach, the diagnostic accuracy of using the dPCR cBRAF V595E assay as a surrogate assay vs. the histologic diagnosis as the gold standard was 85.7% with a 92.3% positive predictive value and an 80.0% negative predictive value (Supplementary Table 2). A small proportion of samples, regardless of the matrix and method of DNA purification, tested positive with less than 1% of positive cells. To rule out the possibility of false positive results in these cases, 7 of these cases were repeated five times on separate days. Six samples consistently tested positive. One sample gave one positive and four negative results. It was considered to be positive with regard to the LOD estimation in order to be more conservative in terms of analytical sensitivity; however, it was considered to be negative in the prospective cohort study.

### Effect of Matrices, Purification Methods and Amplification Methods

**Figure 4:**
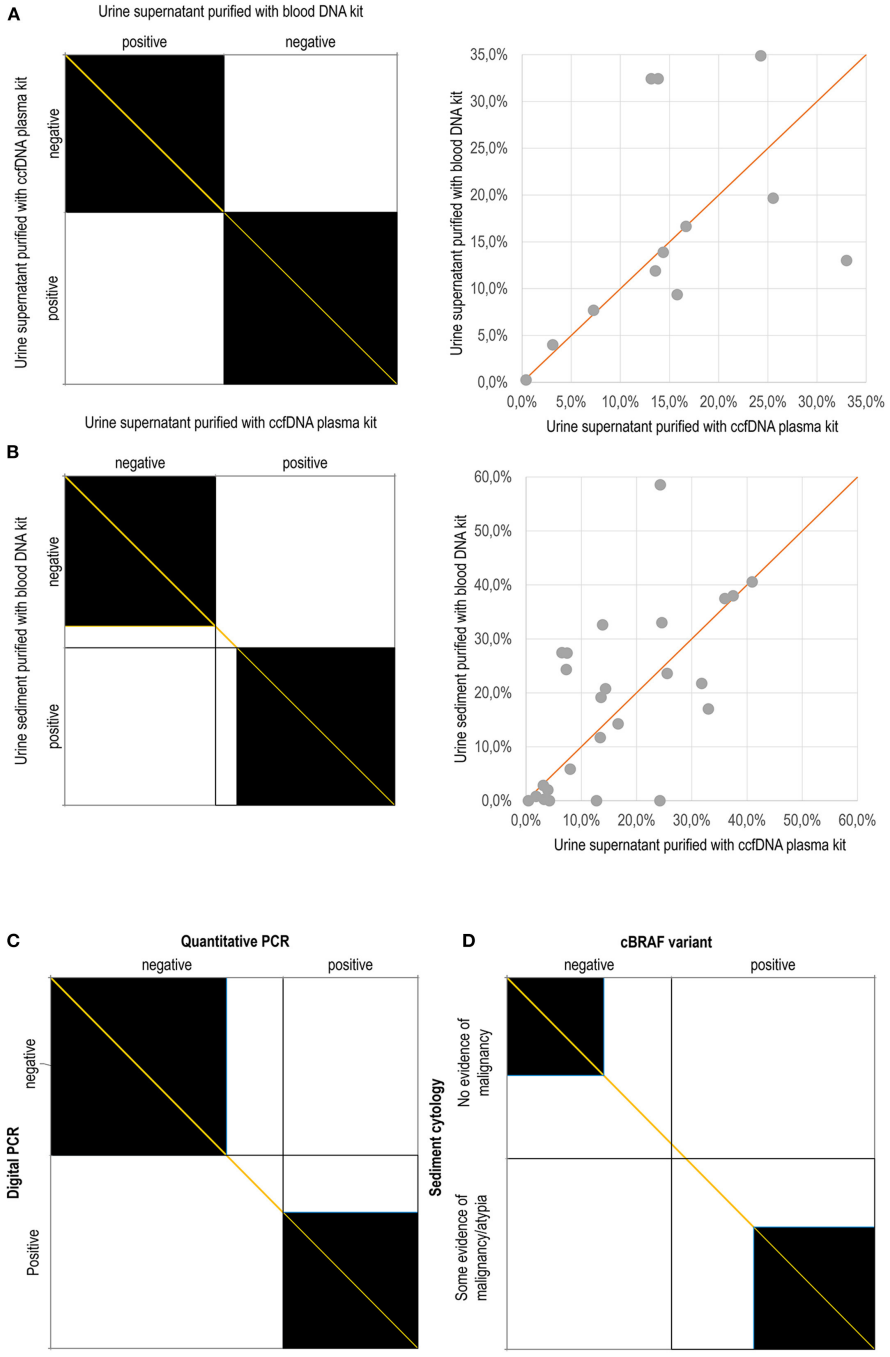
Agreement charts according to Bangdiwala and Shankar (23): The agreement chart provides a visual representation for comparing the concordance in paired categorical data. Agreement is determined by the size of the box. Lesser agreement is visualized by comparing the area of the blackened squares to the area of the rectangles. The direction of method bias is reviewed by examining the “path of the rectangles” and how it deviates from the diagonal line of no bias (orange). (A) Complete agreement between the findings obtained using purification with the Blood DNA kit and the ccfDNA plasma kit on urine supernatant; **(B)** Agreement between the findings obtained using the Blood DNA kit on urine sediment and the ccfDNA plasma kit on urine supernatant; **(C)** Agreement between the findings obtained using digital PCR and quantitative PCR; **(D)** Agreement between the cytological examination of urine sediment and the molecular detection of the cBRAF variant. In dot plot graphs, the percentages refer to the amount of mutated allele with respect to the wild-type allele.

In order to achieve the best diagnostic performance, factors affecting the cBRAF V595E detection were evaluated. In the small subset of samples used to compare the two methods of DNA purification (Blood vs. CCF) in urine supernatant, the preliminary 23 results matched perfectly, and the comparison was discontinued (Figure 4A). The overall accuracy Confidence Interval was between 85.2 and 100.0%. However, surprisingly, when the matrix and the purification methods were compared, the sediment purified using the WB kit and the supernatant purified with CCF showed that the latter was slightly more sensitive. Overall, the accuracy of the WB applied on the urine sediment was 93.5% (CI 82.1-98.6%, Supplementary Material). The agreement plots comparing the findings using either different matrix or purification methods and the scatterplot comparing the quantitative estimate of the BRAF variant whenever it was present were graphed in Figure 4.

The qPCR was highly specific but variably less sensitive than dPCR in detecting the BRAF pathogenic variant, depending on the matrix used (Table 1; Figure 4C). In fact, it performed better on the urine supernatant and sediment than it did in detecting the variant in the FFPE samples. The sensitivity, negative predictive value and, overall, accuracy were 75.0, 78.9, and 87.0% in the urine supernatant, 71.4, 76.2, and 85.1% in the urinary sediments, 52.6, 64.0, and 74.3% in the FFPE samples, respectively (Table 1).

**Table 1:** Results of the comparison between quantitative PCR and digital PCR as gold standards.

**Matrix**	**Parameter**	**Value**	**95% CI**
Urine supernatant	Sensitivity	75.0%	53.3–90.2%
	Specificity	100.0%	84.6–100.0%
	Positive likelihood ratio		
	Negative likelihood ratio	0.25	0.1–0.5
	Positive predictive value	100.00%	
	Negative predictive value	78.57%	64.7–88.0%
	Accuracy	86.96%	73.7–95.1%
Urinary sediment	Sensitivity	71.4%	53.7–85.4%
	Specificity	100.00%	84.56–100.00%
	Positive likelihood ratio		
	Negative likelihood ratio	0.3	0.2–0.5
	Positive predictive value	100.00%	
	Negative predictive value	76.2%	65.5–84.4%
	Accuracy	85.1%	74.3–92.6%
FFPE samples	Sensitivity	52.6%	28.9–75.6%
	Specificity	100.0%	84.6–100.0%
	Positive likelihood ratio		
	Negative likelihood ratio	0.5	0.3–0.8
	Positive predictive value	100.00%	
	Negative predictive value	64.0%	52.5–74.1%
	Accuracy	74.3%	56.7–87.5%

*The parameters of diagnostic performances are indicated and their respective 95% confidence Interval (C.I.). CI, Confidence Interval; FFPE, Formalin-Fixed Paraffin-Embedded*.

### Concordance Between Pathological Diagnosis and BRAF Analysis

Although it is self-evident that histopathology and BRAF mutation testing identify different features of a disease, i.e., the morphological and molecular features, similarly it is self-evident that the latter may support the histopathological diagnosis whenever a carcinoma carries the BRAF variant. As such, the percentage of cases was evaluated whenever both the morphological and the molecular findings supported the presence of malignancy. In the second blind-label cohort, overall concordance was consistent in 85.7% of cases whereas 10.7% of cases had a histologic diagnosis of malignancy without the BRAF mutation, and 3.6% had a histologic diagnosis of a benign neoplasm and the BRAF mutation. The “concordance” was consistent and statistically significant (*p* < 0.001). With regards to the third cohort of samples (urine specimens), the cBRAF variant was detected in 55.3% of cases. Similarly, in 51.3% of the samples, cytological examination allowed the samples to be categorized as having evidence of malignancy with different degrees of likelihood (from possible through likely and very likely).

Regarding the urine findings, the comparison is more complex. For statistical purposes, the cytological diagnoses, were additionally dichotomously clustered into “no evidence of malignancy” including unrewarding findings, inflammation, haematuria, and pyuria, and “some evidence of malignancy/atypia” including epithelial atypia (including dysplasia), likely neoplasia, and neoplasia categories. In this cohort, the cytological findings were suggestive of malignancy in 48.7% (37 out of 76) of cases and did not identify evidence to suggest malignancy in the remaining 51.3% (39 out of 76) of cases. Similarly, the BRAF variant was detected in 55.3% of cases (42 out of 76). However, “concordance” (evidence of cellular atypia with the presence of the BRAF variant and no evidence of malignancy without the presence of the BRAF mutation) was 59.2% (45 out of 76) and was not significant (*p* = 11).

Overall, 40.8% (31 out of 76) of cases were not coincident (Figure 4D). The samples showing discordant results were 22.4% cBRAF positive cases in which neoplastic cells were not detected. The absence of neoplastic cells could be the result of true absence (lack of exfoliation) or sample deterioration. Moreover, 18.4% of the cases classified as having some evidence of malignancy had a negative BRAF result. When this group was additionally narrowed to exclude those cases with generic evidence of atypia/dysplasia without overt evidence of malignancy, then discordant findings were found in only 8 of the 76 cases (10.5%).

## Discussion

The primary aim of this diagnostic cohort study was to assess the analytical performance of a novel dPCR assay, exploiting chip-based partitioning for detecting the BRAF pathogenic variant V595E. Some preanalytical and analytical factors affecting the accuracy in detecting the cV595E BRAF variant in canine urine were then investigated in order to establish a liquid biopsy protocol.

As expected, the dPCR assay showed high accuracy. When diluted in a solution containing wild-type gDNA, a clearly linear relationship was assessed at least up to 0.1%. Similar results confirming high accuracy, were obtained when the analytical sensitivity was assessed in terms of absolute counts by diluting the samples containing the BRAF pathogenic variant in molecular grade water. The LOD was approximately 1 × 10^−4^ mutated target among wild-type targets. The LOD was similar to the ddPCR assay employing slightly different primers and probes (14), much higher than the 10% reported for the Restriction Fragment Length Polymorphism (RFLP) and Sanger Sequencing (8, 14) methods and higher than the 0.1% reported in allele-specific qPCR and targeted next generation sequencing (NGS) (8, 20). High precision was also demonstrated in inter-assay repeatability when the tests were carried out on separate days. These findings confirmed the unparalleled ability of the dPCR technique in detecting very rare variants regardless of the partitioning method (21, 24–31). In a previous study, ddPCR outperformed the Sanger sequencing method in detecting the BRAF mutation in tissue samples (14). Furthermore, the advantage of Sanger Sequencing is that it is not sequence specific, but is warranted in the case of hot-spots where it can detect all the possible different somatic mutations occurring in certain loci. However, Sanger Sequencing is inherently not sensitive enough to detect somatic mutations in cancers below the 10–20% threshold, and it is progressively being replaced by other alternative and more sensitive techniques, such as NGS (8), qPCR (29), and dPCR (14). In fact, differences exist between different dPCR platforms although they tend to be related to throughput and cost rather than to analytical performance.

It should be emphasized that the reported frequency of UC carrying BRAF pathogenic variants also depends on the method used for detection, ranging from 44.6% when assessed by qPCR (15) to 79% of cases when assessed by ddPCR (14) and 87.9% when assessed by targeted NGS (8). The present findings in samples with a histologic diagnosis of UC is very close to the 87.9% BRAF positive UC described in the original study by Decker et al. who used the Amplicon enrichment NGS technique (8) which included a larger cohort of histologically diagnosed samples. The differences can be attributed to the different cohort composition/case selection and/or to the different analytical techniques used and/or to other technical considerations, such as the matrix used for gDNA purification.

In this study, both qPCR and dPCR were carried out on the same samples, and their accuracy was directly compared. As expected, the comparison showed that qPCR was not as reliable (32) as dPCR in detecting the BRAF mutation. When using dPCR as a reference, the qPCR was highly specific but not sufficiently sensitive. As indirect evidence, the prevalence of BRAF mutation assessed by Grassinger et al. (32) which relied on a qPCR using the same primers and probes as in this study, was considerably lower than in other studies. In particular, the present study found that, in FFPE samples, qPCR showed an even lower sensitivity of 52.6% with an inadequate low negative predictive value of 64%. The evidence that qPCR is less accurate than dPCR in analyzing gDNA purified from FFPE tissue is counter-intuitive. However, it may be due to the fact that qPCR is inherently unable to detect the rarer, usually mutated, target and this inability is exacerbated when the DNA concentration or the wild-type “background” is elevated as occurs, for instance, in FFPE derived gDNA samples.

From the perspective of precision medicine, a liquid biopsy approach, relying on a reliable cBRAFV595E variant detection assay, would have therapeutic implications. In this instance, one may require the dPCR BRAF assay in addition to a diagnosis reached using histological examination to decide whether to use a BRAF inhibitor. In this regard, it should be noted that BRAF inhibitors are being evaluated for use in dogs (19), and it is possible that they will soon be part of the veterinary oncologist armory.

The second advantage of liquid biopsy would be to reinforce the diagnosis of UC by exploiting the role of such mutations as a main driver of carcinogenesis and its high prevalence. As such, the mutation is found in the majority of canine urinary bladder and prostate carcinomas, arising from the urothelium and not in benign canine urothelial lesions. However, the possibility that false positive results might arise due to the presence of a BRAF variation in benign lesions should be additionally investigated. In fact, BRAF V600E mutations have been found in human benign melanocytic nevi, (33) and in benign colorectal polyps (34, 35). In this study, in 1 out of 12 benign lesions (a case of prostatic hyperplasia), a positive cBRAFV595E result was found. It was not possible to further investigate the finding of a BRAF mutation in a histologically benign lesion. Additional investigation into BRAF mutations occurring in benign canine urothelial lesions is needed. However, the high frequency of BRAF mutations in canine UC has suggested that the detection of the BRAF pathogenic variant in a clinical sample may corroborate a diagnosis of malignancy. In particular, the dPCR will be of great benefit as a test for improving the accuracy of minimally invasive medical procedures, such as urine collection. This is because UCs arise at inaccessible anatomic sites, making biopsy sampling difficult and invasive. As a minimally invasive alternative, the cytological examination of urinary sediment is warranted. In the presence of clinical suspicion (i.e., bladder mass, haematuria in absence of inflammation), the presence of transitional cells with malignant features, such as a high nuclear-to-cytoplasmic ratio, variable cell and nuclear size, clumped chromatin with prominent nucleoli, and mitotic activity are suggestive of UC. However, inflammation and reactive changes may result in similar cytologic features to neoplastic epithelial cells and it is not always possible to differentiate between them. As a consequence, a definitive diagnosis using urine cytology alone may not be achievable in all cases (36, 37). In these instances, the potential of corroborating a diagnosis of UC by detection of the BRAF pathogenic variant as a surrogate marker of malignancy would be a breakthrough. The assay could also be utilized as a screening assay in high-risk animals, such as predisposed breeds, or for monitoring for the presence of minimal residual disease and for early detection of recurrence. In previous studies, urine reliability was evaluated using dPCR with both oil-water emulsion partitioning (ddPCR) and Sanger Sequencing; good results were obtained, but with some bottlenecks in terms of sensitivity and quality of the samples (14, 15). The reliability of either urine supernatant or sediment, irrespective of the method of collection and of further preservative treatment, was evaluated in this study. To that end, a direct comparison using gDNA purified from FFPE tissues, urine supernatant or urine sediment was carried out regardless of the quantity and quality of the gDNA purified. Interestingly, it was found that the BRAF assay showed a 100% sensitivity when assessing urine compared with other more invasive specimens. Decker et al. (8) found that a less sensitive method, such as RFLP, gave a moderately high concordance (89%) between urine sediment DNA and matched tumor samples, and a 100% concordance when the urine sediment was examined using the more sensitive targeted NGS. Differently from others (8, 14) both the supernatant and the sediment were evaluated being the cell integrity necessary for purifying gDNA from urinary tract cells difficult to be ensured when shipping samples to external labs. The supernatant has the advantage to accumulate a mix of long (> 500bp) gDNA from non-malignant cells and from malignant cells of the urinary tract as well as very short circulating cell-free DNA either filtered from kidneys (20, 38) or arising from apoptotic or necrotic urinary tract malignant cells. This latter has been demonstrated to be critically affected by long-term (1 week) storage at room temperature while the former lasts beyond one week (38). In this study, the supernatant, when the DNA was purified using the CCF methods intended for the liquid biopsy, was shown to be slightly more sensitive than the sediment when the DNA was purified using a standard method (rsc Blood). Conversely, the use of purification methods designed for liquid biopsy seems not to give a substantial advantage over a more standard technique when the urinary supernatant was used. Tumor DNA released in urine is highly fragmented, even more so than circulating cell-free DNA, and ranges from 40 to 250 bp (38). This size of DNA fragments could be lost by standard purification methods. A more specific, detailed and accurate DNA fragment size analysis should be carried out to assess why the CCF purification method did not confer greater advantages. Since the collection procedure was intentionally not standardized to mirror real “field” conditions, it is likely that the purification method might be critical only in a minority of cases characterized by inadequate conservation, repeated freezing-thawing or the presence of nucleases or other factors affecting the integrity of the DNA while in the majority of cases, the two methods are overlapping. Additional studies are needed to address this interesting finding.

When cytological examination and BRAF variant analysis were compared, the coincidental findings were relatively low. A substantial number of samples were identified in which the cytological smear examination detected cellular atypia, but the BRAF assay was negative. This could have been due to the inclusion of all the cases having cytologic evidence of atypia or dysplasia, as “likely neoplastic” for the purposes of statistical analysis, when they occurred as part of a non-neoplastic reactive process. It should be noted that the discordant findings were much lower when only those cases with overt evidence of malignancy were considered. Similarly, there were samples in which molecular testing identified the BRAF pathogenic variant in the urine of cases without cytological evidence of atypia. In many cases, the cytological evaluation of the urinary sediment was inconclusive due to low cellular yield, sample deterioration or atypia relating to inflammation and dysplasia (37). Inclusion of these samples in the statistical analysis as non-neoplastic likely impacted this finding. Samples obtained directly from a catheter have greater success in predicting malignancy (37). However, urine samples with inflammation, pyuria and/or haematuria could potentially mask evidence of malignancy on cytology.

The data presented herein suggest that detection of BRAF in urine could improve the diagnosis of canine UC, particularly in cases in which no definitive cytologic diagnosis was possible and a bladder mass was present. Additional study is clearly warranted to evaluate this exciting possibility.

In conclusion, the present study described a liquid biopsy protocol for the accurate detection of the V595E variant in dogs. The protocol included purification from urine supernatant using the CCF plasma kit intended for very small length DNA and analysis by means of a digital PCR assay. If applied as an ancillary tool to urine cytology, this protocol could improve the diagnostic workout of UC in dogs.

## Data Availability Statement

The original contributions presented in the study are included in the article/Supplementary Material, further inquiries can be directed to the corresponding author/s.

## Ethics Statement

The animal study was reviewed and approved by the Ethics Review Board, the Committee for Animal Welfare, of the University of Bologna. The Committee considers that this type of project does not fall under the legislation for the protection of animals used for scientific purposes, national decree-law 26/2014. The research aims, layout and methods, and all the other aspects mentioned herein were evaluated by the University of Bologna Ethics Committee (COBA) and formally approved under protocol ID 147351. Written informed consent for participation was not obtained from the owners because only leftover samples from routine diagnostics were used in the study.

## Author Contributions

MT and FG: conceptualization. MT, CP, JM, RT, TF, and MN: data curation. FG, MT, CP, JM, TF, and RT: investigation. FS, JM, and FS: supervision. FG and MT: writing—original draft. CP, JM, RT, TF, MN, and FS: writing—review and editing. All authors have read and agreed to the published version of the manuscript.

## Conflict of Interest

CP, MN, JM, and FS are employed by IDEXX Laboratories Ltd. TF is employed by Private Veterinary Clinic San Marco Srl. MT is employed by Genefast srl. The remaining authors declare that the research was conducted in the absence of any commercial or financial relationships that could be construed as a potential conflict of interest.

## Publisher's Note

All claims expressed in this article are solely those of the authors and do not necessarily represent those of their affiliated organizations, or those of the publisher, the editors and the reviewers. Any product that may be evaluated in this article, or claim that may be made by its manufacturer, is not guaranteed or endorsed by the publisher.
