# Supplementary material for: Validation of a Liquid Biopsy Protocol for Canine BRAFV595E Variant Detection in Dog Urine and Its Evaluation as a Diagnostic Test Complementary to Cytology
Source: Front Vet Sci. 2022 May 31;9:909934. doi: 10.3389/fvets.2022.909934 (PMC9195143; doi:10.3389/fvets.2022.909934)
Supplement: Supplementary file 1 [file Data_Sheet_1.docx]

Supplementary Material

# Supplementary Data: detailed methods of quantitative PCR and digital PCR

The qPCR assays were carried out on CFX Connect or CFX96 Touch thermal cyclers (Biorad) using a mixture containing 1x of master mix (TaqMan Universal PCR Master Mix, ThermoFisher Scientific), 1x of primer mix made of forward primer (5’-CATGAAGACCTCACAGTAAAAATAGGTGAT-3’), reverse primer (5’-TGGGACCCACTCCATCGA-3’) and dual-labelled probes (VIC TAGCCACAGTGAAATC – wild-type allele; FAM CCACAGAGAAATC – mutated allele; TaqMan SNP Genotyping assay, Thermo Fisher), 2 µL of gDNA template and molecular biology grade water to reach a final volume of 20 µL. The PCRs were carried out using a 2-step protocol: initial denaturation at 95°C for X min followed by 45 cycles at 95°C for 30 s, 60°C for 45 s. The dPCRs were carried out on the QuantStudio 3D Digital PCR system (Thermo Fisher). The reaction mix is loaded on a chip (QuantStudio 3D Digital PCR 20K Chip Kit v2), the chip is sealed and placed in the Flex 2 × Flat PCR System for thermal cycling. At the end, the chip is inserted into the chip reader (QuantStudio 3D Digital PCR instrument) and analysed using the QuantStudio 3D AnalysisSuite Software. The setup of the dPCR is more straightforward. As with any PCR assay, the main factors to be modified are annealing temperature and length of time of any step. Indeed, there is a greater lag with respect to qPCR since the thermal cycler has to transfer heat to a chip and not a tube. As a general rule, the length of the steps should be 2 minutes. The dPCRs were carried out using a mixture containing 1x of master mix (QuantStudio 3D Digital PCR Master Mix v2; Thermo Fisher Scientific, Monza, Italy), 1x of primer mix (TaqMan SNP Genotyping assay, Thermo Fisher), 2 µL of gDNA template and molecular biology grade water to reach a final volume of xx 15 µL. The PCRs were carried out using a 3-step protocol: initial denaturation at 95°C for 10 min followed by 40 cycles at 95°C for 30 s, 60°C for 2 min and 71° C for 2 min. Firstly, data quality of each 20K chip were made. Quality assessment includes visualization allowing the inspection, clustering of each fluorophore, and copy number calculation. In particular, only chips with at least 17 500 wells correctly read were considered adequate and analysed.

The dPCR should be ideally 100% efficient. The efficiency could be estimated by diluting the target and assessing the linearity. To that end, two experiments were performed: in the first experiment, the target was diluted 1:10 in molecular biology grade water. The expected result was a 1:10 reduction of the target amount in case of optimal efficiency and a reduction of 10 magnitude of the % of BRAF mutated target. In the second experiment, we diluted 1:10 the gDNA purified from a BRAF mutated sample in wild-type gDNA purified from blood. In this case we specifically tested the efficiency of the probe for the mutated allele; hence we expected a 90% reduction of the percentage of the mutated target.
