# Supplementary material for: Validation of a Liquid Biopsy Protocol for Canine BRAFV595E Variant Detection in Dog Urine and Its Evaluation as a Diagnostic Test Complementary to Cytology
Source: Front Vet Sci. 2022 May 31;9:909934. doi: 10.3389/fvets.2022.909934 (PMC9195143; doi:10.3389/fvets.2022.909934)
Supplement: Supplementary file 2 [file Table_1.DOCX]

Supplementary Material

# Suppl. Table 1: Selected cohorts of the study

| ID | cohort | open-label vs blind-label | original diagnosis | Cytology Categorized diagnosis 1 | Cytology Categorized diagnosis 2 | matrix | BRAF V595E |
| --- | --- | --- | --- | --- | --- | --- | --- |
| 1SM | 1 | open-label | TCC |  |  | FFPE/urine | Detected |
| 2SM | 1 | open-label | TCC |  |  | FFPE/urine | Detected |
| 3SM | 1 | open-label | TCC |  |  | FFPE/urine | Detected |
| 4SM | 1 | open-label | TCC |  |  | FFPE/urine | Detected |
| 5SM | 1 | open-label | TCC |  |  | FFPE/urine | Detected |
| 6SM | 1 | open-label | TCC |  |  | FFPE/urine | Detected |
| 7SM | 1 | open-label | TCC |  |  | FFPE/urine | Detected |
| 8SM | 1 | open-label | TCC |  |  | FFPE/urine | Detected |
| 9SM | 1 | open-label | TCC |  |  | FFPE/urine | Detected |
| 10SM | 1 | open-label | TCC |  |  | FFPE/urine | Detected |
| 11 | 2 | blind-label | Bladder carcinoma |  |  | FFPE | Detected |
| 12 | 2 | blind-label | Bladder carcinoma |  |  | FFPE | Detected |
| 13 | 2 | blind-label | Bladder carcinoma |  |  | FFPE | Detected |
| 14 | 2 | blind-label | Bladder carcinoma |  |  | FFPE | Detected |
| 15 | 2 | blind-label | Bladder dysplasia |  |  | FFPE | Not detected |
| 16 | 2 | blind-label | Bladder polyp |  |  | FFPE | Not detected |
| 17 | 2 | blind-label | Bladder polyp |  |  | FFPE | Not detected |
| 18 | 2 | blind-label | Bladder polyps |  |  | FFPE | Not detected |
| 19 | 2 | blind-label | Bladder TCC |  |  | FFPE | Detected |
| 20 | 2 | blind-label | Bladder TCC |  |  | FFPE | Detected |
| 21 | 2 | blind-label | Bladder TCC |  |  | FFPE | Detected |
| 22 | 2 | blind-label | Chronic cystitis |  |  | FFPE | Not detected |
| 23 | 2 | blind-label | Cystitis |  |  | FFPE | Not detected |
| 24 | 2 | blind-label | Cystitis |  |  | FFPE | Not Detected |
| 25 | 2 | blind-label | Eosinophilic polypoid cystitis |  |  | FFPE | Not detected |
| 26 | 2 | blind-label | Eosinophilic polypoid cystitis |  |  | FFPE | Not detected |
| 27 | 2 | blind-label | Metastatic carcinoma |  |  | FFPE | Detected |
| 28 | 2 | blind-label | Nasal adenocarcinoma |  |  | FFPE | Detected |
| 29 | 2 | blind-label | Nasal TCC |  |  | FFPE | Detected |
| 30 | 2 | blind-label | Penile Squamous cell carcinoma |  |  | FFPE | Detected |
| 31 | 2 | blind-label | Polypoid cystitis |  |  | FFPE | Not detected |
| 32 | 2 | blind-label | Prostate hyperplasia |  |  | FFPE | Detected |
| 33 | 2 | blind-label | Prostatic carcinoma |  |  | FFPE | Detected |
| 34 | 2 | blind-label | Prostatic carcinoma |  |  | FFPE | Not detected |
| 35 | 2 | blind-label | Prostatic carcinoma |  |  | FFPE | Detected |
| 36 | 2 | blind-label | PUNLMP |  |  | FFPE | Not detected |
| 37 | 2 | blind-label | TCC |  |  | FFPE | Detected |
| 38 | 2 | blind-label | TCC |  |  | FFPE | Not detected |
| 39 | 2 | blind-label | Urothelial carcinoma |  |  | FFPE | Not detected |
| 40 | 2 | blind-label | V invasive bladder TCC |  |  | FFPE | Detected |
| 41 | 2 | blind-label | V invasive bladder TCC |  |  | FFPE | Detected |
| 42 | 3 | blind-label | Possible Tumor | LT | Tumor likely | Urine sediment/supernatant | Not detected |
| 43 | 3 | blind-label | Hematuria / inflammation | H / I | No evidence | Urine sediment/supernatant | Not detected |
| 44 | 3 | blind-label | Probable prostatic carcinoma | LT | Tumor likely | Urine sediment/supernatant | Not detected |
| 45 | 3 | blind-label | Probable prostatic carcinoma | LT | Tumor likely | Urine sediment | Not detected |
| 46 | 3 | blind-label | Unrewarding | U | No evidence | Urine sediment | Detected |
| 47 | 3 | blind-label | TCC bladder | T | Tumor likely | Urine sediment | Not detected |
| 48 | 3 | blind-label | Unrewarding | U | No evidence | Urine sediment | Detected |
| 49 | 3 | blind-label | Hematuria / bacteriuria | H / I | No evidence | Urine sediment/supernatant | Not detected |
| 50 | 3 | blind-label | Hematuria / cystitis | H / I | No evidence | Urine sediment/supernatant | Detected |
| 51 | 3 | blind-label | Prostatic abscess/cyst/epithelial proliferation | I | No evidence | Urine sediment | Not detected |
| 52 | 3 | blind-label | Hematuria | H | No evidence | Urine sediment/supernatant | Detected |
| 53 | 3 | blind-label | Probable carcinoma | LT | Tumor likely | Urine sediment/supernatant | Detected |
| 54 | 3 | blind-label | Hematuria | H | No evidence | Urine sediment/supernatant | Detected |
| 55 | 3 | blind-label | Probably urothelial proliferation | LT | Tumor likely | Urine sediment/supernatant | Detected |
| 56 | 3 | blind-label | Unrewarding | U | No evidence | Urine sediment/supernatant | Not detected |
| 57 | 3 | blind-label | Pyuria / bacteriuria / poorly preserved epithelial cells | I | No evidence | Urine sediment/supernatant | Detected |
| 58 | 3 | blind-label | Inflammation / poorly preserved epithelial cells | I | No evidence | Urine sediment/supernatant | Detected |
| 59 | 3 | blind-label | Epithelial neoplasia / suspected TCC | T | Tumor likely | Urine sediment/supernatant | Not detected |
| 60 | 3 | blind-label | Pyuria / bacteriuria | I | No evidence | Urine sediment | Detected |
| 61 | 3 | blind-label | Suspect carcinoma | LT | Tumor likely | Urine sediment | Detected |
| 62 | 3 | blind-label | Epithelial atypia | LT | Tumor likely | Urine sediment/supernatant | Detected |
| 63 | 3 | blind-label | Epithelial atypia | LT | Tumor likely | Urine sediment/supernatant | Not detected |
| 64 | 3 | blind-label | History of bladder mass | U | No evidence | Urine sediment/supernatant | Not detected |
| 65 | 3 | blind-label | Epithelial atypia with necrosis | LT | Tumor likely | Urine sediment/supernatant | Not detected |
| 66 | 3 | blind-label | Unrewarding | U | No evidence | Urine sediment/supernatant | Not detected |
| 67 | 3 | blind-label | Hematuria / bacteriuria | H / I | No evidence | Urine sediment/supernatant | Detected |
| 68 | 3 | blind-label | Unrewarding | U | No evidence | Urine sediment/supernatant | Not detected |
| 69 | 3 | blind-label | Pyuria / bacteriuria | I | No evidence | Urine sediment/supernatant | Not detected |
| 70 | 3 | blind-label | Suspected TCC | LT | Tumor likely | Urine sediment/supernatant | Detected |
| 71 | 3 | blind-label | Suspected epithelial neoplasm | LT | Tumor likely | Urine sediment/supernatant | Detected |
| 72 | 3 | blind-label | Polypoid cystitis / possible TCC | LT | Tumor likely | Urine sediment | Not detected |
| 73 | 3 | blind-label | Unrewarding | U | No evidence | Urine sediment/supernatant | Not detected |
| 74 | 3 | blind-label | Hematuria / epithelial atypia | H / LT | Tumor likely | Urine sediment/supernatant | Detected |
| 75 | 3 | blind-label | Unrewarding | U | No evidence | Urine sediment/supernatant | Not detected |
| 76 | 3 | blind-label | Likely urothelial carcinoma | LT | Tumor likely | Urine sediment/supernatant | Detected |
| 77 | 3 | blind-label | Pyuria / Low cellularity | I | No evidence | Urine sediment | Detected |
| 78 | 3 | blind-label | Cellular atypia | LT | Tumor likely | Urine sediment/supernatant | Detected |
| 79 | 3 | blind-label | Unrewarding | U | No evidence | Urine sediment/supernatant | Not detected |
| 80 | 3 | blind-label | Hematuria / epithelial atypia | H LT | Tumor likely | Urine sediment/supernatant | Detected |
| 81 | 3 | blind-label | Hematuria / epithelial atypia | H LT | Tumor likely | Urine sediment/supernatant | Not detected |
| 82 | 3 | blind-label | Hematuria | H | No evidence | Urine sediment/supernatant | Detected |
| 83 | 3 | blind-label | Hematuria / epithelial atypia. | H LT | Tumor likely | Urine sediment/supernatant | Detected |
| 84 | 3 | blind-label | Epithelial dysplasia. | LT | Tumor likely | Urine sediment/supernatant | Detected |
| 85 | 3 | blind-label | Unrewarding | U | No evidence | Urine sediment/supernatant | Detected |
| 86 | 3 | blind-label | Unrewarding | U | No evidence | Urine sediment/supernatant | Not detected |
| 87 | 3 | blind-label | Epithelial atypia | LT | Tumor likely | Urine sediment/supernatant | Detected |
| 88 | 3 | blind-label | Urothelium not malignant | U | No evidence | Urine sediment/supernatant | Not detected |
| 89 | 3 | blind-label | Marked neutrophilic inflammation | I | No evidence | Urine sediment/supernatant | Not detected |
| 90 | 3 | blind-label | Very likely urothelial carcinoma (large nested) | T | Tumor likely | Urine sediment | Detected |
| 91 | 3 | blind-label | Unrewarding | U | No evidence | Urine sediment | Detected |
| 92 | 3 | blind-label | Epithelial atypia / Possible TCC | LT | Tumor likely | Urine sediment | Detected |
| 93 | 3 | blind-label | Neutrophilic inflammation | I | No evidence | Urine sediment | Not detected |
| 94 | 3 | blind-label | Unrewarding | U | No evidence | Urine sediment/supernatant | Not detected |
| 95 | 3 | blind-label | Probable TCC prostate | LT | Tumor likely | Urine sediment/supernatant | Detected |
| 96 | 3 | blind-label | Hematuria | H | No evidence | Urine sediment/supernatant | Not detected |
| 97 | 3 | blind-label | Epithelial atypia. | LT | Tumor likely | Urine sediment/supernatant | Detected |
| 98 | 3 | blind-label | Epithelial atypia. | LT | Tumor likely | Urine sediment/supernatant | Detected |
| 99 | 3 | blind-label | Suspected epithelial atypia | LT | Tumor likely | Urine sediment/supernatant | Detected |
| 100 | 3 | blind-label | Hematuria. | H | No evidence | Urine sediment | Not detected |
| 101 | 3 | blind-label | Hematuria | H | No evidence | Urine sediment | Detected |
| 102 | 3 | blind-label | Epithelial dysplasia / possible carcinoma. | LT | Tumor likely | Urine sediment | Not detected |
| 103 | 3 | blind-label | Likely urothelial carcinoma | LT | Tumor likely | Urine sediment/supernatant | Detected |
| 104 | 3 | blind-label | Urothelial dysplasia / suspect carcinoma. | LT | Tumor likely | Urine sediment | Not detected |
| 105 | 3 | blind-label | Possible low grade TCC | LT | Tumor likely | Urine sediment | Not detected |
| 106 | 3 | blind-label | Likely polypoid cystitis | I | No evidence | Urine sediment | Not detected |
| 107 | 3 | blind-label | Possible TCC clinically | LT | Tumor likely | Urine sediment | Detected |
| 108 | 3 | blind-label | Pyuria / bacteriuria | I | No evidence | Urine sediment | Detected |
| 109 | 3 | blind-label | Epithelial atypia | LT | Tumor likely | Urine sediment | Detected |
| 110 | 3 | blind-label | Epithelial atypia | LT | Tumor likely | Urine sediment | Not detected |
| 111 | 3 | blind-label | Unrewarding | U | No evidence | Urine sediment | Detected |
| 112 | 3 | blind-label | Epithelial atypia | LT | Tumor likely | Urine sediment | Detected |
| 113 | 3 | blind-label | Unrewarding | U | No evidence | Urine sediment | Not detected |
| 114 | 3 | blind-label | Epithelial atypia | LT | Tumor likely | Urine sediment | Detected |
| 115 | 3 | blind-label | Epithelial atypia | LT | Tumor likely | Urine sediment | Detected |
| 116 | 3 | blind-label | Pyuria / Hematuria | H / I | No evidence | Urine sediment | Detected |
| 117 | 3 | blind-label | suspect TCC | LT | Tumor likely | Urine sediment | Not detected |

# TCC: transitional cell carcinoma; PUNLMP: papillary urothelial neoplasm of low malignant potential; FFPE: formalin-fixed paraffin-embedded; LT: likely tumor; U: unrewarding; H: hematuria; I: inflammation; T: likely tumor
