# Supplementary material for: Validation of a Liquid Biopsy Protocol for Canine BRAFV595E Variant Detection in Dog Urine and Its Evaluation as a Diagnostic Test Complementary to Cytology
Source: Front Vet Sci. 2022 May 31;9:909934. doi: 10.3389/fvets.2022.909934 (PMC9195143; doi:10.3389/fvets.2022.909934)
Supplement: Supplementary file 3 [file Table_2.DOCX]

Supplementary Material

# Supplementary Table 2: A) Accuracy of the optimized liquid biopsy protocol versus histopathology (considered gold standard); B) Accuracy of dPCR on DNA purified with WB from urine sediment vs DNA purified using CCF from urine supernatant.

**A**

| **matrix** | **parameter** | **value** | **95% C.I.** |
| --- | --- | --- | --- |
| FFPE (histology) and Urine supernatant (liquid biopsy) | Sensitivity | 80.0% | 51.9% --- 95.7% |
|  | Specificity | 92.3% | 64.0% --- 99.8% |
|  | Positive Likelihood Ratio | 10.4 | 1.6 --- 69.5 |
|  | Negative Likelihood Ratio | 0.22 | 0.1 --- 0.6 |
|  | Positive Predictive Value | 92.3% | 64.2 --- 94.8 |
|  | Negative Predictive Value | 80.0% | 59.0% --- 91.8% |
|  | Accuracy | 85.7% | 67.3% --- 96.0% |

**B**

| **matrix** | **parameter** | **value** | **95% C.I.** |
| --- | --- | --- | --- |
| Urine supernatant | Sensitivity | 88.0% | 68.8% --- 97.4% |
|  | Specificity | 100.0% | 83.9% --- 100.0% |
|  | Positive Likelihood Ratio |  |  |
|  | Negative Likelihood Ratio | 0.12 | 0.0 --- 0.3 |
|  | Positive Predictive Value | 100.0% |  |
|  | Negative Predictive Value | 87.5% | 70.8% --- 95.3% |
|  | Accuracy | 93.5% | 82.1% --- 98.6% |
